# Comparison of Radon and Thoron Concentration Measuring Systems Among Asian Countries

**DOI:** 10.3390/ijerph16245019

**Published:** 2019-12-10

**Authors:** Miroslaw Janik, Shinji Tokonami, Kazuki Iwaoka, Naregundi Karunakara, Shetty Trilochana, Mandya Purushotham Mohan, Sudeep Kumara, Indaje Yashodhara, Weihai Zhuo, Chao Zhao, Fangdong Tang, Linfeng He, Supitcha Chanyotha, Chutima Kranrod, Darwish Al-Azmi, Osamu Kurihara

**Affiliations:** 1 Center for Advanced Radiation Medicine, National Institutes for Quantum and Radiological Science and Technology (QST), Chiba 263-8555, Japan; iwaoka.kazuki@qst.go.jp (K.I.); kurihara.osamu@qst.go.jp (O.K.); 2 Institute of Radiation Emergency Medicine, Hirosaki University, Hirosaki 036-8560, Japan; tokonami@hirosaki-u.ac.jp; 3 Centre for Advanced Research in Environmental Radioactivity (CARER), Mangalore University, Karnataka 574199, India; drkarunakara@gmail.com (N.K.); 3loshetty@gmail.com (S.T.); mohanmp6@gmail.com (M.P.M.); sudeepphy@gmail.com (S.K.); iyashu84@gmail.com (I.Y.); 4 Institute of Radiation Medicine, Fudan University, Shanghai 200032, China; whzhuo@fudan.edu.cn; 5 Shanghai Institute of Measurement and Testing Technology (SIMT), Shanghai 201203, China; zhaoc11@fudan.edu.cn (C.Z.); tangfd@simt.com.cn (F.T.); helf@simt.com.cn (L.H.); 6 Natural Radiation Survey and Analysis Research Unit (NRSA-RU), Chulalongkorn University, Bangkok 10330, Thailand; supitchacu18@gmail.com (S.C.); kranrodc@gmail.com (C.K.); 7 Department of Applied Sciences, College of Technological Studies, Public Authority for Applied Education and Training, Shuwaikh, PO Box 42325, Kuwait City 70654, Kuwait; dalazmi@yahoo.co.uk

**Keywords:** radon, thoron, comparison

## Abstract

Comparison is an important role in the quality control and quality assurance for any measuring system. Due to the future legal regulations regarding radon levels in the air, maintaining the system quality and harmonization of results as well as validation of radon and thoron measuring systems is important. The aim of this work is to validate the degrees of equivalence and measurement precisions of the existing five radon and four thoron measuring systems located in four Asian countries (China, India, Japan and Thailand) through comparison experiment. In this project, comparison experiment was performed in order to derive the ratio between assigned value obtained from one transfer measurement device for radon and one transfer measurement device for thoron belongs to National Institutes for Quantum and Radiological Science and Technology and participants’ value from their measuring instrument. As a result, the ratio value associated with measurement uncertainty was derived for each activity concentration. Finally, measurement bias and degrees of equivalence between the assigned values and values of measurement quantity from participants’ measuring instruments were statistically analysed and presented.

## 1. Introduction

Inhalation of radon (${}^{222}$Rn), hereafter referred to as Rn, thoron (${}^{220}$Rn) hereafter referred to as Tn and their progenies has the highest contribution of the natural ionizing radiation exposure to humans. Hence, in order to protect the public by reducing their exposure to Rn, the European Union lays down maximum reference level for Rn concentrations in indoor air to 300 Bq m${}^{-3}$ [1]. In Asian countries, the legal regulations or recommendations regarding indoor Rn levels in dwellings or workplaces were established in China, Korea and Australia.

However, the metrological traceability such as of Rn (Tn) concentration measurements requires calibration and periodical check with respect to measurement standard of Rn (Tn) which should be performed using calibration-measuring systems equipped with accurate reference measuring instrument and carried out in well-defined measurement condition. Hence, the precision and linearity of the reference measuring instruments and the metrological compatibility of measurement results are essential to ensure the reliability of Rn (Tn) concentration measurement.

In this study, two objectives were investigated: (1) evaluation of the metrological compatibility of Rn (Tn) concentration measurement standards among Asian countries; (2) assessment of the precision and linearity of participants’ measuring instruments response at different Rn (Tn) activity concentrations.

## 2. Materials and Methods

### 2.1. Comparison Procedure

The comparison of measurand atmospheres can be implemented in two ways—(1) by sending the participants’ measuring instruments to a designated laboratory in order to obtain proper exposure, then the instruments (after being exposed) are sent back to the participants’ for further data analysis or (2) alternatively, the organizer participant can send the transfer measuring device to each individual laboratory for measurements (with pre-arranged conditions) and then gather all the data for inter-comparison and details analysis.

In our presented work, the second option has been chosen. Two transfer measuring devices, one for Rn (AlphaGUARD, Bertin GMBH, Germany) and one for Tn (RTM2200, Sarad GMBH, Germany), were selected and referred here as the Transfer Measuring Devices (TMDs). The data registered by TMDs were used for the calculations of performance statistics, between the participants’ Reference Measuring Instruments (referred as RMI) and TMD for each measuring system.

The Rn TMD from the National Institutes for Quantum and Radiological Science and Technology (QST), referred in this work as RnTMD has already been calibrated in a secondary Rn standard laboratory with reference to the Federal Office for Radiation Protection (BfS) and the Physikalisch-Technische Bundesanstalt (PTB) in Germany and in the National Institute of Standards and Technology (NIST) in USA as well as in the National Physical Laboratory (NPL) in the UK. It was exposed in participants’ Rn measuring system for a comparison with their RMIs. Whereas in the case of Tn, the TMD instrument from QST (TnTMD) referred to the manufacturer calibration only. Therefore, such a Tn device was checked in the QST Tn measuring system using the commercially an available Natural Rock Sample [2]. The background values of TMDs were determined at QST using nitrogen gas.

Five laboratories for Rn and four for Tn participated in the exercise conducted during the period from November 2017 to December 2018. The TMDs devices were exposed in participants’ measuring systems at two to three different activity concentrations (low, middle, high) depending on the limiting operation conditions in each individual laboratory within the range of 500–5000 Bq m${}^{-3}$ for Rn and 300–13,000 Bq m${}^{-3}$ for Tn. Similar procedure for Rn was carried out in Europe described elsewhere [3].

### 2.2. Characteristics of Participant’s Facilities

In general, the Rn concentration measuring system (consisting of Rn chamber, Rn source and measuring devices) may work in three modes—(1) static; (2) semi-dynamic; and (3) dynamic.

In the static mode, Rn is injected at once to the tightly closed chamber and then Rn concentration level decreases due to the natural radioactive decay as well as leakage. In the semi-dynamic mode, further Rn injections are introduced to the chamber in order to maintain the required level of Rn concentration. Furthermore, in the dynamic mode, Rn is injected continuously into the Rn chamber with a controlled flow rate and proper air exchange in order to maintain the required concentration level of Rn inside the chamber.

In the presented exercise, one of the Rn-measuring systems is working in static mode, one in semi-dynamic mode and the other three are in dynamic mode. Three of Rn chambers were made of stainless steel, one of plexiglass and one of glass. Their volumes varied from 0.1 to 25 m${}^{3}$. The range of achievable Rn concentrations are from 340–20,000 Bq m${}^{-3}$, depending on the measuring system operation condition. Some of the parameters such as humidity and temperature may be controlled, whereas pressure cannot. The time to reach stable or starting Rn concentration level varies from 1 to 36 h depending on the measuring system construction as well as the Rn source.

Four laboratories are equipped with Tn-measuring systems. Due to the short half-life of Tn, of 55.6 s, the volumes of Tn chambers are smaller than those for Rn; in the range from 0.05 to 0.22 m${}^{3}$ and all of them are working in dynamic mode. Because of different type of sources and set-up procedures at each laboratory, time to achieve stable Tn concentration is different, varying from 0.2 to 20 h. Humidity can be controlled in all measuring system, temperature in two of them and pressure in none of them. Only one laboratory uses calibrated Tn source and three of them use their own made-sources. In two laboratories, the reference measuring instruments have been calibrated at PTB with reference Tn atmosphere, whereas in the other two the calibration was performed using Monte Carlo simulations. The detailed characteristics of the five Rn and the four Tn-measuring systems are presented in Table 1 and Table 2.

### 2.3. Characteristics of Devices

Rn transfer measurement device (RnTMD) provided by QST and reference measuring instruments used in LAB1 (RnRMI1), LAB2 (RnRMI2), LAB4 (RnRMI4) as well as LAB5 (RnRMI5) laboratories are of the same type and based on the use of ionization chamber with 0.56 litre of active volume. Advantage of it is stability in time, low uncertainty, low influence of humidity and linearity of response from 2 Bq m${}^{-3}$ to 2 MBq m${}^{-3}$ of Rn concentration measurement provided by the manufacturer. According to the manual, the system linearity uncertainty is less than 3% for *k* = 1 (note: when the data represent a normal distribution, the *k* factor reflects the number of standard deviations used when calculating a confidence level; for example, *k* = 1 represents an uncertainty of 1 standard deviation and approximately a 68% confidence level) and instrument calibration uncertainty is within ±3% for *k* = 1 (plus uncertainty of the primary standard). It can work in either diffusion or flow mode. In this experiment the manufacturer calibration of TMD was used, which is secondary calibration to BfS accreditation laboratory [7,8]. LAB3 used device (RnRMI3) is equipped with approx. 1 litre chamber with high electric field and Passivated Implanted Planar Silicon (PIPS) to attract the positively-charged polonium daughters, ${}^{218}$Po (T${}_{1/2}$ = 3.10 min; E${}_{\alpha }$ = 6.00 MeV) and ${}^{214}$Po (T${}_{1/2}$ = 164 μs; E${}_{\alpha }$ = 7.67 MeV) which are then counted as a measure of the Rn activity in air. For faster analyses, the ${}^{218}$Po is preferred; as it will reach radioactive equilibrium with Rn in only about 15 min (${}^{214}$Po requires about 3 h for equilibrium because of the intermediate ${}^{214}$Pb and ${}^{214}$Bi daughters). In contrast to the Rn device (RnRMI1,2,4,5) described earlier, this device is working only in the flow mode with the nominal 1 litre min${}^{-1}$ flow rate. The measurement range of this monitor is 4.0 Bq m${}^{-3}$ to 0.75 MBq m${}^{-3}$ within 5% measurement accuracy as described in the manual. Declared calibration uncertainty for this devices is within ±3% with *k* = 2.

The principle of operation of the Tn transfer measurement device (TnTMD) is similar to that of RnRMI3 device. The difference is that this measuring instrument uses four chambers with four ion-implemented silicon detectors (one detector in each chamber) unlike of one detector chamber with one PIPS detector. The total internal volume is smaller than RnRMI3 and it is 250 mm${}^{3}$. This measuring instrument is working with flow mode only with nominal flow rate of 0.3 litre min${}^{-1}$ but it can be regulated by dedicated software. TnTMD used manufacturer calibration but it may be additionally checked by using the Natural Rock Sample system consists of 600 g sample of granite gravel from a stone quarry.

TnRMI1, TnRMI2 and TnRMI4 are devices of similar type as RnRMI3. On the other hand, LAB3 uses measuring device (TnRMI3) based on scintillation counter and particularly Lucas cell type (self-developed with cylindrical one). Accumulated gas is filtered out of the progeny through a special filter and then the radioactive decay is counted. The inside of the scintillation cell is coated with ZnS(Ag)—a chemical that emits light when struck by alpha particles. A photomultiplier tube at the top of the scintillation cell counts the photons and sends the count to a data logger [9]. The TnRMI3 device range of Tn concentration measurement for 60 min cycle is between 30 Bq m${}^{-3}$ to 20 MBq m${}^{-3}$ with relative expanded uncertainty of 10% for *k* = 2. Data logger collects measured counts and dedicated software compensate Tn progeny growing in time. The response of this device was simulated using Monte Carlo method and compared to the measuring instrument calibrated at the PTB [10].

### 2.4. Evaluation Metrics

Several rating schemes have been developed for determining a laboratory’s performance and the meaning of the results of the different scoring systems are not always comparable. Among various statistics, bias, *z*-scores and *E${}_{n}$*-scores are most often used and calculated using following formulas described in References [11,12]. The first stage in producing of a score for comparing results from all participants is obtaining the estimate ratio factor expressed as follow:

(1) $$Ratio=\frac{C_{TMD}^{\prime }}{C_{RMI}^{\prime }}=\frac{k_{TMD}\;\left(C_{TMD}-C0_{TMD}\right)}{k_{RMI}\;\left(C_{RMI}-C0_{RMI}\right)},$$

where:

$C_{TMD}^{\prime }$—the assigned value, that is, the value of the measurand which is the average of Rn (Tn) concentration measured in participants measuring system using RnTMD (TnTMD) calculated as corrected value of measured by TMD included calibration factor and background data [Bq m${}^{-3}$]

$k_{TMD}$—calibration factor of TMD [-]

$C_{TMD}$—measured concentration by TMD [Bq m${}^{-3}$]

$C0_{TMD}$—background concentration of TMD [Bq m${}^{-3}$]

$C_{RMI}^{\prime }$—the laboratory expected value, that is, participant average Rn (Tn) concentration which is corrected value of measured by RMI included calibration factor and background data [Bq m${}^{-3}$]

$k_{RMI}$—calibration factor of participant RMI [-]

$C_{RMI}$—measured concentration by participant RMI [Bq m${}^{-3}$]

$C0_{RMI}$—background concentration of participant RMI [Bq m${}^{-3}$].

To evaluate the bias of the reported results, the relative bias (RB) between participant’s expected value and assigned value is calculated and expressed as a percentage:

(2) $$RB\;\left[\%\right]=\frac{C_{RMI}^{\prime }-C_{TMD}^{\prime }}{C_{TMD}^{\prime }}\times 100\%,$$

The *z*-scores is frequently advised as a performance indicator. The drawback of *z*-scores is that uncertainty of the TMD measurement result is not taken into account for the evaluation of performance and is calculated in accordance to the following equation:

(3) $$z=\frac{C_{RMI}^{\prime }-C_{TMD}^{\prime }}{\sigma_{pt}},$$

where $\sigma_{pt}$ is the standard deviation for proficiency assessment (SDPA) of measurand given by each laboratory determined from the robust standard deviation of participant results (*note:* robust statistics is a way of summarising results as more resistant to the statistical influences of outlying events in a sample population—hence the term “robust”. There are several schemes to asses SDPA but in our case it was calculated using the “median absolute deviation” (MAD) [13,14,15]).

In the case of $E_{n}$-scores, the evaluation includes uncertainties of the participant measurements and the uncertainty of the TMD results and is given by:

(4) $$E_{n}=\frac{\left|C_{RMI}^{\prime }-C_{TMD}^{\prime }\right|}{\sqrt{U_{RMI}^{2}+U_{TMD}^{2}}}.$$

where:

$U_{RMI}$—the participant extended standard uncertainty (*k* = 2)

$U_{TMD}$—the assigned value extended standard uncertainty (*k* = 2).

$E_{n}$-scores can be used in conjunction *z*-scores, to improve the performance of participant by assessing the acceptability of the results using absolute values of $E_{n}$-scores and *z*-scores ($\left|z\right|$ values). The criteria used to assess the performance of each laboratory were $\left|z\right|\leq$ 2 and $E_{n}$≤ 1, satisfactory; 2 $<\left|z\right|<$ 3, questionable; $\left|z\right|\geq$ 3 and $E_{n}$> 1, unsatisfactory.

## 3. Results and Discussion

### 3.1. Results for Rn

The Rn tests were performed over quite a wide range of environmental parameters: temperature from 19 ${}^{\circ }$C to 26 ${}^{\circ }$C, air pressure from 990 hPa to 1030 hPa and relative humidity from 7 to 59%.

It was decided to carry out experiments in Rn-measurement systems at three different concentration levels, that is, Exp1 ≈ 500, Exp2 ≈ 2500, Exp3 ≈ 5000 Bq m${}^{-3}$. Laboratory 2 omitted highest exposure level due to its limiting operation conditions (see Table 1). TMD and RMIs were exposed to each concentration level over 12 h. The measurement interval was set-up at 10 or 60 min depending on participant’s standard exposure procedure.

The results of the Rn experiments are listed in Table 3. Rn concentrations measured by participants ranging from 500 to 5000 Bq m${}^{-3}$ with expanded uncertainty (*k* = 2) in the range from 2 to 9%.

**Figure 1:**
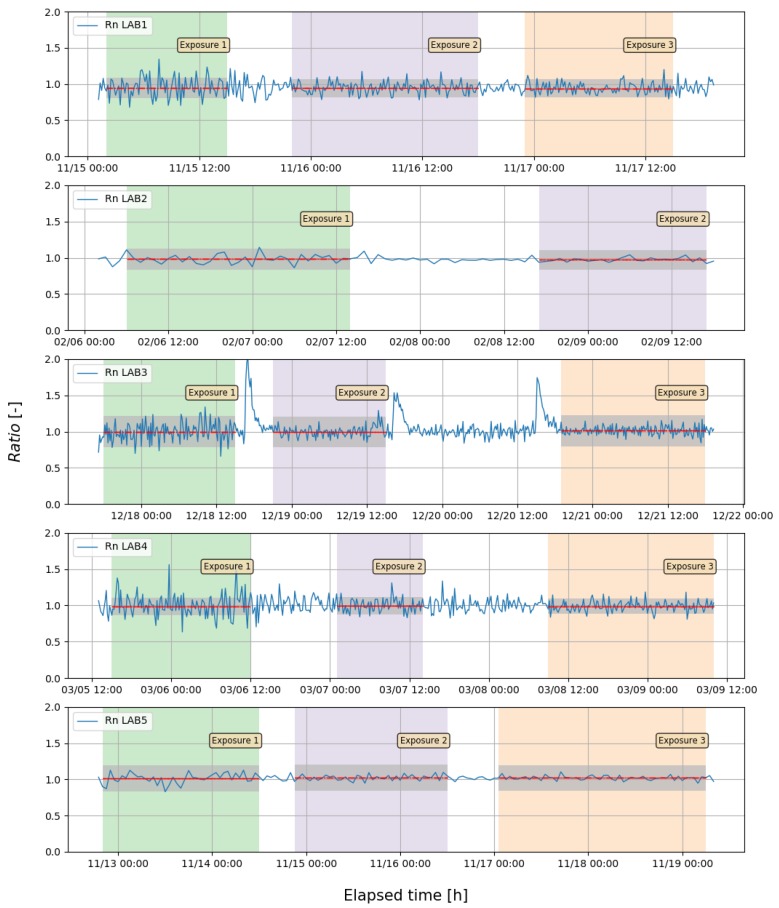
Ratio of Rn concentration (solid line) with mean value for each exposure (dashed line) and expanded uncertainty with *k* = 2 (grey area).

**Figure 2:**
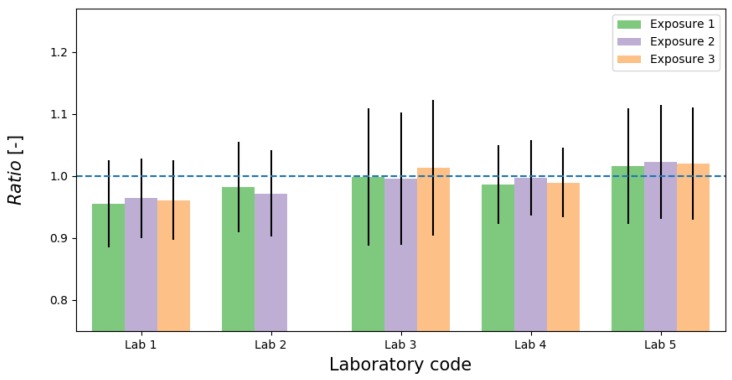
Barplot of Rn Ratio with expanded uncertainty (*k* = 2) against exposure level.

The assessment of results was given by evaluation metrics described in Section 2.4. The ratios of TMD/RMI in each measurement point for each laboratory and exposure are presented in Figure 1. The individual ratios fluctuated but the average ratio of Rn concentration registered by RnTMD at all exposure levels to that of concentration of participants ranged from 0.95 to 1.02, with *RB* metric of −2 to 5% (Figure 2). It means that the difference within laboratories do not across 7%. Highest bias values were registered for measuring system in LAB1 worked in semi-dynamic mode. It should be noted that for all results the uncertainty of ratio are stable in whole range of concentration, what it can be connected to dominant of calibration factors uncertainties in total uncertainty budget. Moreover, experiment in LAB1 was performed in variable Rn conditions because of natural Rn source where the stable concentration cannot be obtained (semi-dynamic mode). Experiments in LAB2, LAB4 and LAB5 were performed with stable Rn concentration (dynamic mode), whereas in LAB3 in the static mode.

All results of *E${}_{n}$*-scores were <1 and it suggest that measuring systems fulfilled the satisfactory conditions. In addition, all z-scores metric values were in the first range of acceptance level, i.e., $\left|z\right|\leq$ 2.

### 3.2. Results for Tn

**Figure 3:**
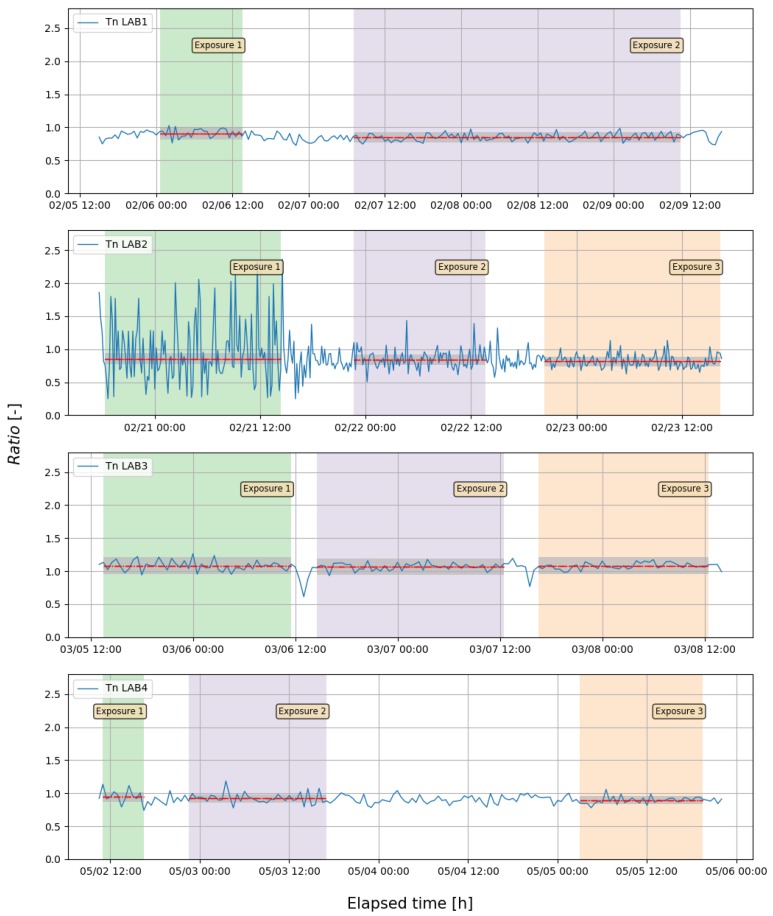
Ratio of Tn concentration (solid line) with mean value for each exposure (dashed line) and expanded uncertainty with *k* = 2 (grey area).

**Figure 4:**
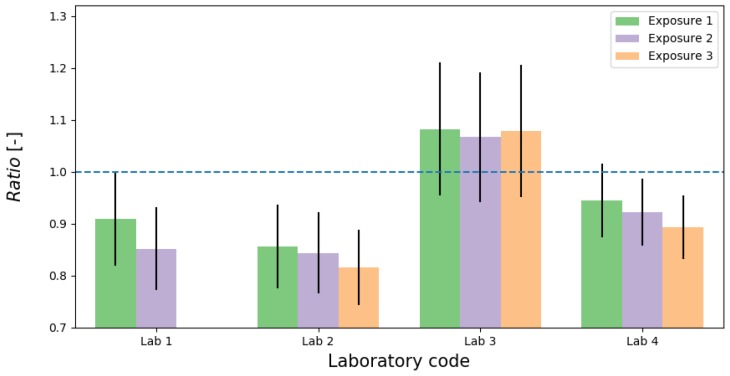
Barplot of Tn results (*Ratio*) with expanded uncertainty (*k* = 2) against exposure level.

The results of Tn experiment are summarized in Table 4 and presented in Figure 3 and Figure 4. Tn concentrations were determined as the average concentrations in the participants’ measuring system for 5.5–51.5 h exposure time with stable Tn conditions. Average Tn concentration registered by TnTMD was in the range from 300 to 13,000 Bq m${}^{-3}$ with expanded uncertainty (*k* = 2) from 3 to 10%. In contrast to Rn, evaluation of Tn consist only ratio and relative bias calculated for each laboratory and exposure, because of TnTMD was not traceable to primary standard. As mentioned earlier, the goal of this Tn exercise was to show the discrepancies in Tn measurements among different laboratories and indicate potential sources of those differences.

The Ratio with uncertainty of Tn concertation registered by TnTMD to Tn concentration registered by participants’ TnRMI for each exposure are showing in Figure 4 and ranged from 0.82 to 1.08, with RB metric range from −8 to 23%. It means that the largest difference within laboratories is 31%.

The results presented in Figure 4 and Table 4 shown that LAB3 maintained a stable *Ratio* and *RB* values whereas LAB1, LAB2 and LAB4, which used the same measuring instrument, were giving an downward trend of *Ratio* versus concentration. These causes of phenomena for this measuring instrument might come from several factors such as problem with assessment of conversion factor, measuring instrument type and construction, measuring instrument sensitivity, the difference in the experimental conditions, such as humidity, temperature, different Tn standard source and so forth.

### 3.3. Uncertainty Budget

In a Rn (Tn) comparison experiment, the main elements of uncertainty are associated with: calibration factors, background and activity concentration registered by the RMIs and TMDs.

Examples of an uncertainty budget assessment for Rn and Tn are presented in Table 5 and Table 6. It can be observed that for Rn the highest contributions comes from the calibration factors of reference measuring instruments and transfer measurement devices and the measurement results, whereas for Tn values calibration factors uncertainty dominant. The background has no contribution to the total uncertainty budget.

## 4. Conclusions

The Rn and Tn intercomparison experiments, using one reference device for Rn and one for Tn, were performed at five Rn and four Tn laboratories. The results for Rn by all laboratories reveal a very good agreement with each other within the percentage bias less or equal 5% to values provided by Transfer Measurement Device.

Tn results showed Tn measurement problems with linearity related to Tn behaviour, measuring instrument or calibrations therefore further studies are needed to explain phenomena that cause these differences.

It can be concluded that these experiments provide important information for quality of Rn and Tn measuring systems among Asian countries.

## Figures and Tables

**Table 1:** Characteristic of Rn measuring systems and measuring instruments along with chamber volume [m${}^{-3}$], achievable Rn range [Bq m${}^{-3}$], time to reach stable Rn condition [h], environmental parameters: pressure [hPa], temperature [${}^{\circ }$C], relative humidity [%], Rn traceability, primary measuring instrument for Rn measurement and chamber operation mode.

Institution	LAB1	LAB2	LAB3	LAB4	LAB5
Chamber volume [Bq m${}^{-3}$]	22.7	0.54	0.1	3.8	25
Achievable Rn range[Bq m${}^{-3}$]	500–15,000	340–3600	1000–10,000	370–20,000	500–8000
Time to reach stable
Rn condition [h]	1	36	3.5	1.5	12
Environmental parameters					
Pressure [hPa]	Ambient	Ambient	Ambient	Ambient	Ambient
Temperature [${}^{\circ }$C]	10–50	Ambient	25–30	6–40	5–30
Humidity [%]	30–90	10–55	50–60	10–95	30–90
Rn traceability
(source/institute/etc)	Factory calibration	BfS	Calibrated Rn source	National standard	BfS
Primary measuring
instrument for Rn measurement	RnRMI1	RnRMI2	RnRMI3	RnRMI4	RnRMI5
Operation mode	Semi-dynamic	Dynamic	Static	Dynamic	Dynamic

**Table 2:** Characteristic of Tn measuring systems and measuring instruments along with chamber volume [m${}^{-3}$], achievable Tn range [Bq m${}^{-3}$], time to reach stable Tn condition [h], environmental parameters: pressure [hPa], temperature [${}^{\circ }$C], relative humidity [%], Tn traceability, primary measuring instrument for Tn measurement and chamber operation mode.

Institution	LAB1	LAB2	LAB3	LAB4
Chamber volume [m${}^{-3}$]	0.15	0.05	0.22	0.15
Achievable Tn range[Bq m${}^{-3}$]	3500–30,000	1000–50,000	3500–12,000	3000–30,000
Time to reach stable
Tn condition [h]	20	0.5	0.2	15
Environmental parameters				
Pressure [hPa]	Ambient	Ambient	Ambient	Ambient
Temperature [${}^{\circ }$C]	Ambient	25–30	0–50	Ambient
Humidity [%]	10–60	50–60	10–95	20–60
Tn traceability
(source/institute/etc)	Lucas scintillation cell and Monte Carlo simulation [4,5]	Calibration Tn source	PTB and Monte Carlo simulation [6]	PTB
Primary measuring
instrument for Tn measurement	TnRMI1	TnRMI2	TnRMI3	TnRMI4
Operation mode	Dynamic	Dynamic	Dynamic	Dynamic

**Table 3:** Results of Rn exercise and environmental parameters inside chambers during experiments with uncertainties in parenthesis, where: $C_{RMI}^{\prime }$ is Rn concentration with uncertainty of participant RMI [Bq m${}^{-3}$], $C_{TMD}^{\prime }$ is Rn concentration with uncertainty of TMD [Bq m${}^{-3}$], Time is exposure time [h] and RB is the relative bias [%]. Ratio, RB, $E_{n}$ and *z*-score are evaluation metrics.

	Exposure	$C_{\mathrm{RMI}}^{\prime }$ [Bq m${}^{-3}$]	$C_{\mathrm{TMD}}^{\prime }$ [Bq m${}^{-3}$]	Time [h]	Temperature [${}^{\circ }$C]	Relative Humidity [%]	Pressure [hPa]	Ratio [-]	RB [%]	$E_{n}$ [-]	*z*-Score [-]
LAB1	Exp1	734 ± 38	700 ± 38	13	26.0 ± 0.3 ${}^{a}$	52.2 ± 1.7 ${}^{a}$	996 ± 1 ${}^{b}$	0.95 ± 0.05	5	0.6	0.2
	Exp2	2730 ± 136	2623 ± 136	20	25.9 ± 0.2 ${}^{a}$	52.4 ± 1.6 ${}^{a}$	996 ± 1 ${}^{b}$	0.96 ± 0.04	4	0.6	0.3
	Exp3	4837 ± 241	4634 ± 241	16	26.0 ± 0.2 ${}^{a}$	52.0 ± 1.7 ${}^{a}$	996 ± 1 ${}^{b}$	0.96 ± 0.04	4	0.6	0.3
LAB2	Exp1	775 ± 40	761 ± 40	32	22.5 ± 0.4 ${}^{b}$	6.9 ± 0.3 ${}^{a}$	998 ± 3 ${}^{b}$	0.98 ± 0.04	2	0.2	0.5
	Exp2	2724 ± 138	2648 ± 139	24	22.1 ± 0.6 ${}^{b}$	50.1 ± 1.2 ${}^{a}$	1013 ± 2 ${}^{b}$	0.97 ± 0.04	3	0.4	0.3
LAB3	Exp1	526 ± 48	525 ± 28	21	27.3 ± 0.2 ${}^{b}$	48.8 ± 3.7 ${}^{b}$	1013 ± 1 ${}^{b}$	1.00 ± 0.06	0	0	0
	Exp2	2810 ± 251	2799 ± 146	18	27.2 ± 0.2 ${}^{b}$	53.3 ± 1.8 ${}^{b}$	1014 ± 1 ${}^{b}$	1.00 ± 0.06	0	0	0
	Exp3	4474 ± 398	4534 ± 235	23	27.1 ± 0.5 ${}^{b}$	58.6 ± 1.8 ${}^{b}$	1010 ± 1 ${}^{b}$	1.01 ± 0.06	−1	0.1	0
LAB4	Exp1	520 ± 15	513 ± 27	21	20.8 ± 0.5 ${}^{a}$	18.9 ± 0.5 ${}^{a}$	1022 ± 1 ${}^{b}$	0.99 ± 0.04	1	0.2	0.1
	Exp2	2520 ± 65	2512 ± 132	13	19.6 ± 0.3 ${}^{a}$	20.1 ± 0.3 ${}^{a}$	1023 ± 1 ${}^{b}$	1.00 ± 0.04	0	0.1	0
	Exp3	5001 ± 114	4948 ± 255	25	19.0 ± 1.2 ${}^{a}$	20.6 ± 1.2 ${}^{a}$	1024 ± 1 ${}^{b}$	0.99 ± 0.03	1	0.2	0.2
LAB5	Exp1	611 ± 44	620 ± 33	40	24.0 ± 0.5 ${}^{a}$	35.9 ± 1.2 ${}^{a}$	1010 ± 1 ${}^{b}$	1.02 ± 0.05	−2	0.2	−0.1
	Exp2	2461 ± 177	2518 ± 131	39	23.4 ± 0.2 ${}^{a}$	37.7 ± 0.6 ${}^{a}$	1019 ± 2 ${}^{b}$	1.02 ± 0.05	−2	0.3	−0.6
	Exp3	4803 ± 345	4899 ± 253	53	23.4 ± 0.1 ${}^{a}$	37.9 ± 0.4 ${}^{a}$	1014 ± 2 ${}^{b}$	1.02 ± 0.05	−2	0.2	−0.7

${}^{a}$ controlled, ${}^{b}$ uncontrolled.

**Table 4:** Results of Tn exercise and environmental parameters inside chambers during experiments with uncertainties in parenthesis, where: $C_{RMI}^{\prime }$ is Tn concentration with uncertainty of participant RMI [Bq m${}^{-3}$], $C_{TMD}^{\prime }$ is Tn concentration with uncertainty of TMD [Bq m${}^{-3}$], Time is exposure time [h] and RB is the relative bias [%]. Ratio and RB are evaluation metrics.

	Exposure	$C_{\mathrm{RMI}}^{\prime }$ [Bq m${}^{-3}$]	$C_{\mathrm{TMD}}^{\prime }$ [Bq m${}^{-3}$]	Time [h]	Temperature [${}^{\circ }$C]	Relative Humidity [%]	Pressure [hPa]	Ratio [-]	RB [%]
LAB1	Exp1	4489 ± 440	4083 ± 201	13	19.1 ± 0.3 ${}^{b}$	8.9 ± 0.5 ${}^{a}$	998 ± 1 ${}^{b}$	0.91 ± 0.09	10
	Exp2	7247 ± 710	6175 ± 303	51.5	19.1 ± 0.4 ${}^{b}$	14.3 ± 2.2 ${}^{a}$	1008 ± 4 ${}^{b}$	0.85 ± 0.08	17
LAB2	Exp1	332 ± 33	284 ± 14	20	28.3 ± 0.1 ${}^{b}$	NA ${}^{c}$	1008 ± 1 ${}^{b}$	0.86 ± 0.08	17
	Exp2	3599 ± 353	3037 ± 149	15	28.4 ± 0.1 ${}^{b}$	NA ${}^{c}$	1008 ± 1 ${}^{b}$	0.84 ± 0.08	19
	Exp3	4999 ± 490	4079 ± 200	20	27.6 ± 0.1 ${}^{b}$	NA ${}^{c}$	1010 ± 2 ${}^{b}$	0.82 ± 0.07	23
LAB3	Exp1	3549 ± 348	3843 ± 189	22	20.0 ± 0.2 ${}^{a}$	40.0 ± 0.4 ${}^{a}$	1024 ± 3 ${}^{b}$	1.08 ± 0.13	−8
	Exp2	7005 ± 687	7477 ± 367	22	20.0 ± 0.2 ${}^{a}$	40.0 ± 0.4 ${}^{a}$	1021 ± 2 ${}^{b}$	1.07 ± 0.12	−6
	Exp3	10190 ± 999	10995 ± 539	20	20.0 ± 0.2 ${}^{a}$	40.0 ± 0.4 ${}^{a}$	1021 ± 2 ${}^{b}$	1.08 ± 0.13	−7
LAB4	Exp1	3151 ± 106	2977 ± 150	5.5	28.4 ± 0.1 ${}^{b}$	38.7 ± 0.5 ${}^{a}$	1008 ± 1 ${}^{b}$	0.95 ± 0.07	6
	Exp2	5709 ± 164	5264 ± 258	18.5	26.2 ± 0.2 ${}^{b}$	39.0 ± 0.6 ${}^{a}$	1015 ± 2 ${}^{b}$	0.92 ± 0.06	8
	Exp3	12951 ± 371	11571 ± 568	16.5	27.5 ± 0.3 ${}^{b}$	39.8 ± 0.8 ${}^{a}$	1008 ± 1 ${}^{b}$	0.89 ± 0.06	12

${}^{a}$ controlled, ${}^{b}$ uncontrolled, ${}^{c}$ not available due to technical problem.

**Table 5:** Example of budget calculation for Rn exposure.

Quantity	Value	Uncertainty (*k* = 2)	Contribution
$C_{TMD}^{\prime }$	513.00	27.00	11%
$C0_{TMD}$	3.00	9.00	0%
$k_{TMD}$	0.98	0.05	59%
$C_{RMI}^{\prime }$	520.00	15.00	18%
$C0_{RMI}$	2.00	2.00	0%
$k_{RMI}$	1.08	0.02	12%
Ratio	0.99	0.04	4% (*k* = 2)

**Table 6:** Example of budget calculation for Tn exposure.

Quantity	Value	Uncertainty (*k* = 2)	Contribution
$C_{TMD}^{\prime }$	11,571	568	0%
$C0_{TMD}$	3	5	0%
$k_{TMD}$	1.0	0.05	42%
$C_{RMI}^{\prime }$	12,951	371	0%
$C0_{RMI}$	9	7	0%
$k_{RMI}$	2.03	0.12	58%
Ratio	0.89	0.06	6% (*k* = 2)
